# *Helicobacter pylori *infection and gastroduodenal diseases in Vietnam: a cross-sectional, hospital-based study

**DOI:** 10.1186/1471-230X-10-114

**Published:** 2010-09-30

**Authors:** Tung L Nguyen, Tomohisa Uchida, Yoshiyuki Tsukamoto, Dung T Trinh, Long Ta, Bang H Mai, Song H Le, Ky D Thai, Dung D Ho, Hai H Hoang, Takeshi Matsuhisa, Tadayoshi Okimoto, Masaaki Kodama, Kazunari Murakami, Toshio Fujioka, Yoshio Yamaoka, Masatsugu Moriyama

**Affiliations:** 1 Department of Molecular Pathology, Faculty of Medicine, Oita University, Yufu City (879-5593), Oita Prefecture, Japan; 2 Department of Gastroenterology, Faculty of Medicine, Oita University, Yufu City (879-5593), Oita Prefecture, Japan; 3 Department of Human Environmental and Social Medicine, Faculty of Medicine, Oita University, Yufu City (879-5593), Oita Prefecture, Japan; 4 Department of Pathology, 108 Hospital, No.1 Tran Hung Dao Street, Hanoi City, Vietnam; 5 Department of Gastroenterology, 108 Hospital, No.1 Tran Hung Dao Street, Hanoi City, Vietnam; 6 Department of Molecular Biology, 108 Hospital, No.1 Tran Hung Dao Street, Hanoi City, Vietnam; 7 Department of Endoscopy, Cho Ray Hospital, No.201B Nguyen Chi Thanh Street, Ho Chi Minh City, Vietnam; 8 Department of Training and Research, Cho Ray Hospital, No.201B Nguyen Chi Thanh Street, Ho Chi Minh City, Vietnam; 9 Department of Gastrointestinal Endoscopy, Tama-Nagayama Hospital, Nippon Medical School, Tokyo (113-8602), Japan; 10 Department of Environmental and Preventive Medicine, Faculty of Medicine, Oita University, Yufu City (879-5593), Oita Prefecture, Japan

## Abstract

**Background:**

The rate of *H. pylori *infection in Vietnam is reportedly high, but the spectrum of *H. pylori*-associated gastroduodenal diseases has not been systematically investigated. Moreover, despite the similarities of ethnicity and diet, the age-standardized incidence rate of gastric cancer in the northern city of Hanoi is higher than that in the southern city of Ho Chi Minh, but the reason for this phenomenon is unknown. The virulence of Vietnamese *H. pylori *has also not been investigated in detail.

**Methods:**

Individuals undergoing esophagogastroduodenoscopy were randomly recruited. *H. pylori *infection status was determined based on the combined results of culture, histology, immunohistochemistry, rapid urine test and serum ELISA. Peptic ulcer (PU) and gastroesophageal reflux disease was diagnosed by endoscopy, and chronic gastritis was determined histologically. *H. pylori *virulence factors were investigated by PCR and sequencing.

**Results:**

Among the examined patients, 65.6% were infected with *H. pylori*. The prevalence of infection was significantly higher in those over 40 years of age than in those aged ≤40. Chronic gastritis was present in all *H. pylori*-infected individuals, 83.1% of whom had active gastritis, and 85.3% and 14.7% had atrophy and intestinal metaplasia, respectively. PU was present in 21% of infected patients, whereas its incidence was very low in non-infected individuals. The prevalence of PU was significantly higher in Hanoi than in Ho Chi Minh. The prevalence of *vacA m1*, which has been identified as an independent risk factor for PU in Vietnam, was significantly higher among *H. pylori *isolates from Hanoi than among those from Ho Chi Minh.

**Conclusions:**

*H. pylori *infection is common in Vietnam and is strongly associated with PU, active gastritis, atrophy and intestinal metaplasia. *vacA m1 *is associated with an increased risk for PU and might contribute to the difference in the prevalence of PU and gastric cancer between Hanoi and Ho Chi Minh.

## Background

*Helicobacter pylori *(*H. pylori*) is a spiral, Gram-negative bacterium that chronically infects more than half of the world's population, and is currently recognized to play a causative role in the pathogenesis of gastritis, gastroduodenal ulcer, gastric adenocarcinoma and mucosa-associated lymphoid tissue (MALT) lymphoma [1,2]. Infection with *H. pylori *almost always results in chronic gastritis, but more severe diseases such as peptic ulcer and gastric cancer develop in only a small proportion of infected patients, suggesting that the clinical outcomes are probably determined by the interaction of bacterial virulence, host genetic susceptibility and environmental factors [2,3]. To date, several *H. pylori *virulence factors associated with severe clinical outcomes have been reported, including *cagA*, *cagE*, *vacA*, *babA*, *oipA*, *iceA *and *homB *[4-11].

In Vietnam, the rate of *H. pylori *infection is reportedly high [12], but the spectrum of *H. pylori*-associated gastroduodenal diseases has not been investigated systematically. Moreover, despite the similarities of ethnicity and diet, the age-standardized incidence rate (ASR) of gastric cancer in the northern city of Hanoi is about 1.5 times higher than that in the southern city of Ho Chi Minh (27.0 vs. 18.7 cases per 100.000 males and 13.2 vs. 8.1 cases per 100.000 females, respectively) [13], but the reason for this intriguing phenomenon is unknown. Additionally, the ASR of gastric cancer in Vietnam is approximately 3 times lower than that in Japan and Korea [13], despite the fact that the prevalence of *H. pylori *infection in Vietnam is reportedly higher [12]. This phenomenon, regarded as an "Asian enigma", is thought to be partly attributable to geographic variations in bacterial virulence [14,15]. Nevertheless, the virulence of Vietnamese *H. pylori *strains has not been extensively investigated. Therefore, we carried out the present cross-sectional study to clarify these unresolved issues.

## Methods

### Patients

Individuals undergoing esophagogastroduodenoscopy at the endoscopy centers of two major hospitals in Hanoi and Ho Chi Minh were randomly selected. Local ethics approval and written informed consent from all participants were obtained before the study. Exclusion criteria included a history of partial gastric resection, *H. pylori *eradication therapy and treatment with antibiotics, bismuth-containing compounds, H_2_-receptor blockers or proton pump inhibitors within 1 month before the study. Overall, the study subjects comprised 270 participants (153 females and 117 males) aged 14 to 86 years (mean age, 42.5 years), including 134 from Hanoi and 136 from Ho Chi Minh (Table 1).

**Table 1:** Characteristics of the study population

	*H. pylori-*positive	*H. pylori-*negative	Total
No. of participants (%)	177 (65.6%)	93 (34.4%)	270
Number (%) in region			
Hanoi	89 (66.4%)	45 (33.6%)	134
Ho Chi Minh	88 (64.7%)	48 (35.3%)	136
Sex, no. (%)			
Female	101 (66%)	52 (34%)	153
Male	76 (65%)	41 (35%)	117
Age group, no. (%)			
≤40	67 (57.8%)	49 (42.2%)	116
≤20	6 (66.7%)	3 (33.3%)	9
21-30	24 (53.3%)	21 (46.7%)	45
31-40	37 (59.7%)	25 (40.3%)	62
> 40	110 (71.4%)	44 (28.6%)	154
41-50	62 (71.3%)	25 (28.7%)	87
51-60	30 (69.8%)	13 (30.2%)	43
>60	18 (75.0%)	6 (25.0%)	24
Mean age (range)	43.7 (14-83)	40.2 (18-86)	42.5 (14-86)
Disease			
Peptic ulcer	37	1	38
Gastric cancer	0	0	0
MALT lymphoma	0	0	0
Chronic gastritis	177	68	145
Active	147	11	158
Non-active	30	57	87
Atrophy	151	53	204
Intestinal metaplasia	26	5	31
GERD	4	5	9
Normal	0	25	25

Before endoscopy, participants were interviewed by trained medical staff to ascertain their medical history and lifestyle factors. During endoscopy, 5 biopsy specimens (two from the antrum, two from the corpus and one from the upper part of the lesser curvature) were taken and subsequently used for *H. pylori *culture and histopathologic examination. Peptic ulcer disease (PU) including gastric ulcer (GU), duodenal ulcer (DU) and gastroduodenal ulcer (GDU), and gastroesophageal reflux disease (GERD) were diagnosed by endoscopic observation, while chronic gastritis was determined histologically. After endoscopy, urine and blood samples from all participants were collected on the same day and tested using the rapid urine test and a serum ELISA kit, respectively.

### Determination of *H. pylori *infection status

To maximize the diagnostic accuracy, 5 different methods were combined for the diagnosis of *H. pylori *infection, including culture, histology, immunohistochemistry, rapid urine test and serum ELISA.

For *H. pylori *culture, two biopsy specimens from the antrum and body were homogenized in saline and inoculated onto Mueller Hinton II Agar medium (Becton Dickinson, NJ, USA) supplemented with 7% horse blood without antibiotics. The plates were incubated for up to 10 days at 37°C under microaerophilic conditions (10% O_2_, 5% CO_2 _and 85% N_2_). *H. pylori *was identified on the basis of colony morphology, gram staining and positive reactions for oxidase, catalase, and urease. Isolated strains were stored at -80°C in Brucella Broth (Difco, NJ, USA) containing 10% dimethylsulfoxide and 10% horse serum.

HE and Giemsa staining, and immunohistochemistry with anti-*H. pylori *polyclonal antibody (Dako, Denmark) were performed using serial sections of three specimens from the antrum, corpus and upper part of the lesser curvature, as described previously [16]. Results were judged by an experienced pathologist (T.U.) who was unaware of other information about the subjects.

Urine and blood samples were collected and tested using the urine RAPIRUN test (Otsuka Pharmaceutical Co., Ltd., Tokyo, Japan) and E Plate kit (Eiken Chemical Co., Ltd., Tokyo, Japan), respectively, in accordance with the manufacturers' instructions.

*H. pylori*-positive status was determined by positive culture or, in the case of negative culture, by at least two positive results among the following tests: histology, immunohistochemistry, rapid urine test and serum ELISA. Patients were diagnosed as *H. pylori*-negative when all five tests gave negative results. If only one test other than culture was positive, patients were considered to have undetermined *H. pylori *infection status and were excluded from further analysis.

### Diagnosis and scoring of chronic gastritis

Biopsy specimens were stained with HE and examined by an experienced pathologist (T.U.). For each biopsy specimen, the grades of neutrophil infiltration, mononuclear cell infiltration, atrophy and intestinal metaplasia were scored on the basis of the updated Sydney System (0, none; 1, mild; 2, moderate; and 3, severe).

Active gastritis was classified into four groups as described previously: (i) non-active gastritis, (ii) antrum-predominant gastritis (AP), (iii) corpus-predominant gastritis (CP) and (iv) pangastritis (PAN) [17,18].

### Investigation of *H. pylori *virulence factors

*H. pylori *was subcultured from the stock. Multiple colonies on agar plates were harvested, and genomic DNA was extracted using the standard phenol-chloroform method.

The virulence factors of *H. pylori*, including *cagA*, *cagE*, *vacA s/m/i*, *iceA*, *babA *and *homB*, were examined using PCR as described previously elsewhere [5,7,8,19-22]. The status of *oipA*, "on" or "off", was determined by examining its signal sequence, as described previously [10]. Sequencing was performed with a Big Dye Terminator v3.1 Cycle Sequencing Kit on a ABI Prism 310 Genetic Analyzer (Applied Biosystems, CA, USA) in accordance with the manufacturer's instructions. All the primers used in this study were purchased from Sigma-Aldrich Japan.

### Statistical analysis

Chi-squared test, Ficher's exact test, one-way ANOVA test, and multivariate analysis were used. Differences at *p *< 0.05 were regarded as statistically significant. Data analysis was performed using SPSS statistical software v.16.0 (SPSS Inc., Chicago, USA).

## Results

### Prevalence of *H. pylori *infection

Among the 270 participants, 100, 131, 133, 143 and 141 gave positive results by culture, histology, immunohistochemistry, rapid urine test and serum ELISA, respectively. Based on the combined results of these tests, 177 patients (65.6%) were judged to be infected with *H. pylori*, 93 (34.4%) were *H. pylori*-negative, and none was considered to have undetermined *H. pylori *infection status (Table 1). There was no significant difference in the prevalence of infection between females and males (66.0% vs. 65.0%), or between Hanoi and Ho Chi Minh (66.4% vs. 64.7%) (Table 1). The prevalence of infection did not differ significantly among the 10-year age groups, but was significantly higher in persons over 40 years of age than in those aged ≤40 years (71.4% vs. 57.8%, p = 0.021) (Table 1).

### *H. pylori *infection and gastroduodenal diseases

Among the 270 participants, 38 (14.1%) were endoscopically diagnosed as having PU, including 21 DUs, 6 GUs and 11 GDUs (DU/GU ratio, 3.5); gastric cancer and MALT lymphoma were not detected (Table 1). The prevalence of PUD was 37/177 (20.9%) in the *H. pylori*-infected group but only 1/93 (1.1%) in the non-infected group (Table 1). Multivariate analysis showed that the presence of PU was strongly associated with *H. pylori *infection (adjusted OR, 27.8; 95% CI, 3.6 - 200; p = 0.001), but not with other factors such as sex, age group, alcohol and coffee consumption, and smoking.

The prevalence of PU was 20.1% (27/134) in Hanoi but much lower in Ho Chi Minh, 8.1% (11/136). For *H. pylori*-infected subjects, the prevalence was 30.3% (27/89) and 11.4% (10/88) in Hanoi and Ho Chi Minh, respectively. Multivariate analysis showed that subjects in Hanoi had a higher risk for PU than those in Ho Chi Minh, with an adjusted OR of 2.6 (95% CI, 1.2 - 5.9; p = 0.02) for the overall population and 3.0 (95% CI, 1.3 - 7.0; p = 0.01) for *H. pylori*-infected subjects. Among *H. pylori*-related PU, the proportion of GDU was significantly higher in Hochiminh than in Hanoi. In contrast, the proportion of GU in Hanoi appeared to be higher than that in Hochiminh but this difference did not reach the statistical significance (Table 2).

**Table 2:** Clinical outcomes among *H. pylori*-infected patients in Hanoi and Hochiminh

Disease	Hanoi (n = 89)	Hochiminh (n = 88)	p
Peptic ulcer	27 (30.3%)	10 (11.4%)	< 0.05
Duodenal ulcer	19	2	N.S
Gastric ulcer	6	0	N.S
Gastroduodenal ulcer	2	8	< 0.05
Chronic gastritis	89 (100%)	88 (100%)	N.S
Active gastritis	70 (78.7%)	77 (87.5%)	N.S
Antrum-predominant gastritis	42	59	< 0.05
Corpus-predominant gastritis	3	3	N.S
Pangastritis	25	15	< 0.05
Atrophy	76 (85.4%)	75 (85.2%)	N.S
Intestinal metaplasia	15 (16.9%)	11 (12.5%)	N.S

N.S, not significant

GERD was diagnosed endoscopically in 9 cases (3.3%) and was not inversely associated with *H. pylori *infection status (p = 0.282).

### *H. pylori *infection and chronic gastritis

**Figure 1:**
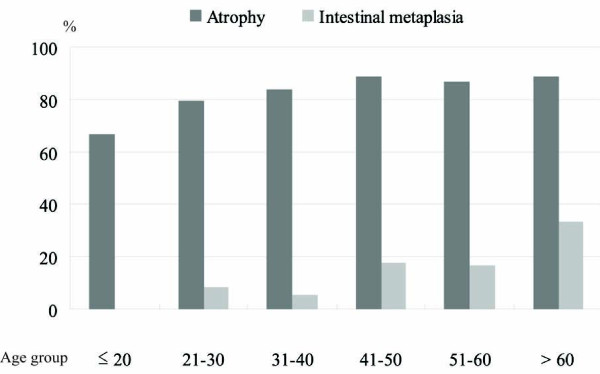
**Prevalence of gastric atrophy and intestinal metaplasia among *H. pylori*-infected Vietnamese by age group**.

Chronic gastritis was present in all *H. pylori*-infected persons, the majority of whom (147/177, 83.1%) had active gastritis, including AP (57.1%), CP (3.4%) and PAN (22.6%). Atrophic gastritis had developed in the majority of them (151/177, 85.3%), even in younger individuals, and its prevalence appeared to increase with age (Figure 1). About 14.7% (26/177) of infected persons had intestinal metaplasia, which was always accompanied by atrophy (Table 1 and Figure 1). The prevalence of antrum-predominant gastritis among *H. pylori*-infected patients was significantly lower in Hanoi than in Hochiminh, whereas the prevalence of pangastritis in Hanoi was significantly higher than that in Hochiminh. However, there was no significant difference in the prevalences of atrophy and intestinal metaplasia between the two cities (Table 2).

Multivariate analysis showed that *H. pylori *positivity was strongly associated with active inflammation (adjusted OR, 41.7; 95% CI, 18.9 - 90.9; p < 0.001), chronic inflammation (adjusted OR, 19.2; 95% CI, 5.5 - 66.7; p < 0.001), atrophy (adjusted OR, 4.2; 95% CI, 2.3 - 7.7; p < 0.001) and intestinal metaplasia (adjusted OR, 3.2; 95% CI, 1.1 - 9.8; p = 0.036) in the gastric mucosa. An age of >40 years conferred an increased risk for atrophy (adjusted OR, 3.0; 95% CI, 1.6 - 5.5; p < 0.001) and intestinal metaplasia (adjusted 3.1; 95% CI, 1.2 - 7.9; p = 0.02).

### *H. pylori *virulence factors and peptic ulcer

An important focus of our interest was why the prevalence of PU was significantly higher in Hanoi than in Ho Chi Minh. It is currently accepted that the clinical outcomes of *H. pylori *infection are determined by the interaction of bacterial virulence, and host and environmental factors. Because the ethnicity, environmental factors and prevalence of *H. pylori *infection in the two regions are similar, we focused on bacterial virulence. We investigated genetic factors of *H. pylori *that are reportedly associated with gastroduodenal diseases, including *cagA*, *cagE*, *vacA (s/m/i)*, *babA*, *oipA*, *iceA*, and *homB*, in 100 clinical strains (53 from Hanoi and 47 from Ho Chi Minh), of which 24 and 76 strains were isolated from patients with PU and chronic gastritis, respectively. Details of the results are presented in Table 3. Multivariate analysis showed that only *vacA m1 *was an independent risk factor for PU (adjusted OR, 3.0; 95% CI, 1.1 - 8.4; p = 0.034). Moreover, infection with *H. pylori *that simultaneously possessed *vacA m1 *together with *cagA*, *cagE *and *babA *(quadruple positivity) conferred an increased risk for PU (OR, 4.2; 95%CI, 1.5 - 11.3, p = 0.005). Interestingly, the prevalences of *vacA m1 *and quadruple positivity were significantly higher among *H. pylori *isolates from Hanoi than among those from Ho Chi Minh (58.5% vs. 36.2%, p = 0.029 and 54.7% vs. 34.0%, p = 0.046, respectively) (Table 4). In addition, comparison of virulence factors of *H. pylori *infecting DU and GU patients revealed no significant difference (data not shown).

**Table 3:** Virulence factors of *H. pylori *from Vietnam

	Total (n = 100)	Peptic ulcer (n = 24)	Chronic gastritis (n = 76)	p
*cagA*				> 0.05
Positive	95 (95%)	24 (100%)	71 (93.4%)	
Negative	5 (5%)	0 (0%)	5 (6.6%)	
*cagE*				> 0.05
Positive	88 (88%)	24 (100%)	64 (84.2%)	
Negative	12 (12%)	0 (0%)	12 (15.8%)	
*vacA m *†				< 0.05
*m1*	48 (48%)	17 (70.8%)	31 (40.8%)	
*m2*	52 (52%)	7 (29.2%)	45 (59.2%)	
*vacA s*				> 0.05
*s1*	100 (100%)	24 (100%)	76 (100%)	
*s2*	0 (0%)	0 (0%)	0 (0%)	
*vacA i*				> 0.05
*i1*	94 (94%)	24 (100%)	70 (92.1%)	
*i2*	6 (6%)	0 (0%)	6 (7.9%)	
*babA*				> 0.05
Positive	92 (92%)	24 (100%)	68 (89.5%)	
Negative	8 (8%)	0 (0%)	8 (10.5%)	
*oipA status*				> 0.05
On	100 (100%)	24 (100%)	76 (100%)	
Off	0 (0%)	0 (0%)	0 (0%)	
*iceA*				> 0.05
*iceA1*	50 (50%)	13 (54.2%)	37 (48.7%)	
*iceA2*	44 (44%)	11 (45.8%)	33 (43.5%)	
Both *iceA1/2*	3 (3%)	0 (0%)	3 (3.9%)	
None	3 (3%)	0 (0%)	3 (3.9%)	
*homB*				> 0.05
Positive	39 (39%)	7 (29.2%)	32 (42.1%)	
Negative	61 (61%)	17 (70.8%)	44 (57.9%)	
Quadruple positivity †				< 0.05
Yes	45 (45%)	17 (70.8%)	28 (36.8%)	
No	55 (55%)	7 (29.2%)	48 (63.2%)	

† Prevalence was significantly different between peptic ulcer and chronic gastritis

**Table 4:** Prevalence of *H. pylori *virulence factors in Hanoi and Ho Chi Minh

	Hanoi (n = 53)	Ho Chi Minh (n = 47)	p (Hanoi vs. Ho Chi Minh)
*cagA*			N.S
Positive	51 (96.2%)	44 (93.6%)	
Negative	2 (3.8%)	3 (6.4%)	
*cagE*			N.S
Positive	49 (92.5%)	39 (83.0%)	
Negative	4 (7.5%)	3 (6.4%)	
*vacA m*			0.034
*m1*	31 (58.%)	17 (36.2%)	
*m2*	22 (41.5%	30 (63.8%)	
*vacA s*			N.S
*s1*	53 (100%)	48 (100%)	
*s2*	0 (0%)	0 (0%)	
*vacA i*			N.S
*i1*	50 (94.3%)	44 (93.6%)	
*i2*	3 (5.7%)	3 (6.4%)	
*babA*			N.S
Positive	50 (94.3%)	42 (89.4%)	
Negative	3 (5.7%)	5 (10.6%)	
*oipA status*			N.S
On	53 (100%)	48 (100%)	
Off	0 (0%)	0 (0%)	
*iceA*			N.S
*iceA1*	26 (49.1%)	24 (51.1%)	
*iceA2*	23 (43.4%)	21 (44.7%)	
Both *iceA1/2*	2 (3.8%)	1 (2.1%)	
None	2 (3.8%)	1 (2.1%)	
*homB*			N.S
Positive	19 (35.8%)	20 (42.6%)	
Negative	34 (64.2%)	27 (57.4%)	
Quadruple positivity			0.046
Yes	29 (54.7%)	16 (34.0%)	
No	24 (45.3%)	31 (66.0%)	

N.S, not significant.

## Discussion

We found that the prevalence of *H. pylori *infection in Vietnam was about 66%, being somewhat similar to that in several other Asian countries [12]. In this study, *H. pylori *infection was proved to be strongly associated with active gastritis, atrophy, intestinal metaplasia, and especially PU (adjusted OR, 27.8; p = 0.001). We did not detect any case of gastric cancer or MALToma, possibly due to their low prevalence and the small sample size. *H. pylori *is probably a very important cause of PU in Vietnam, as PU was observed in approximately 21% of infected patients, while it was virtually absent in non-infected subjects.

We discovered that the majority of *H. pylori *isolates from Vietnam possessed *cagA*, *cagE*, *babA*, *oipA *"on", *vacA s1 *and *vacA i1*, while other genes such as *vacA m1*, *homB *and *iceA1 *were less frequent. Among these virulence factors, only *vacA m1 *was identified as an independent risk factor for PU (adjusted OR, 3.0). Moreover, the co-existence of *vacA m1 *with *cagA*, *cagE *and *babA *conferred an increased risk for PU, perhaps due to a synergic effect among them. Current evidence indicates that the ASR of gastric cancer in Hanoi is about 1.5 times higher than that in Ho Chi Minh [13], and this appeared to accord with the differences between the two cities in the prevalences of antrum-predominant gastritis and pangastritis. Moreover, in the present study the prevalence of PU in Hanoi was also proved to be significantly higher. Our results suggest that the higher prevalence of PU in Hanoi might be attributable to the higher prevalence of *H. pylori *strains carrying *vacAm1 *and quadruple positivity in Hanoi, in comparison with Ho Chi Minh. Because *H. pylori *virulence was associated with both PU and gastric cancer, our data, although not direct, are the first to indicate a reason for the difference in the ASR of gastric cancer between these two regions of Vietnam.

Compared with Japan, the ASR of gastric cancer in Vietnam is much lower [13], and this seems to be reflected by the DU/GU ratio as well as the pattern of chronic gastritis observed in Vietnamese patients. The DU/GU ratio has been regard as a good marker for evaluating the risk of gastric cancer in certain populations because DU patients have low risk of developing gastric cancer whereas the opposite is true for GU patients [18,23]. The DU/GU ratio in Vietnam is about four times higher than that in Japan, but still much lower than that in India, where the ASR of gastric cancer is very low [13,23]. Corresponding well to the high DU/GU ratio, the majority of infected Vietnamese have antrum-predominant gastritis (57%) whereas the rate of corpus-predominant gastritis is low (3.4%) in comparison with Japan (14~17%) [17,18].

Because *H. pylori *virulence is thought to contribute partly to the geographic variation in the ASR of gastric cancer [14,15], we compared various virulence factors of Vietnamese strains with those of Japanese ones. From the literature, it is evident that nearly all Japanese *H. pylori *strains simultaneously carry *cagA, cagE, vacAm1/s1/i1, babA *and *oipA *"on" [6,10,11,22,24], while among Vietnamese strains, this figure is around 45%. Furthermore, the prevalence of *homB*-positive strains is also higher in Japan than in Vietnam (over 90% vs. 39%) [8,25]. From these data, it is tempting to speculate that the difference in ASR of gastric cancer is attributable partly to the difference in bacterial virulence between the two countries.

## Conclusions

Our study indicates that *H. pylori *infection is common in Vietnam and is strongly associated with the development of PU, active gastritis, atrophy and intestinal metaplasia. Moreover, *H. pylori vacA m1 *is associated with an increased risk for PU and might contribute to the difference in the prevalence of PU and gastric cancer between Hanoi and Ho Chi Minh.

## Competing interests

The authors declare that they have no competing interests.

## Financial support

This study was supported in part by a Grant-in-Aid for Young Scientists from the Ministry of Education, Science, Sports and Culture of Japan (B, 227903480003)

## Authors' contributions

TLN and TU carried out the experiments, data analysis and drafting of the manuscript. YT, DTT, LT, BHM, SHL, KDT, DDH, HHH, TM, and TO participated in the collection of patients and contributed to the manuscipt. MK, KM, TF, YY and MM participated in the design, coordination and analysis of the study. All authors read and approved the final manuscript.

## Pre-publication history

The pre-publication history for this paper can be accessed here:

http://www.biomedcentral.com/1471-230X/10/114/prepub

## References

[B1] Peek RM Jr Blaser MJ Helicobacter pylori and gastrointestinal tract adenocarcinomas Nat Rev Cancer 2002 2 28 37 10.1038/nrc703 11902583

[B2] Suerbaum S Michetti P Helicobacter pylori infection N Engl J Med 2002 347 1175 1186 10.1056/NEJMra020542 12374879

[B3] Fox JG Wang TC Inflammation, atrophy, and gastric cancer J Clin Invest 2007 117 60 69 10.1172/JCI30111 17200707 PMC1716216

[B4] Atherton JC Peek RM Jr Tham KT Cover TL Blaser MJ Clinical and pathological importance of heterogeneity in vacA, the vacuolating cytotoxin gene of Helicobacter pylori Gastroenterology 1997 112 92 99 10.1016/S0016-5085(97)70223-3 8978347

[B5] Basso D Zambon CF Letley DP Stranges A Marchet A Rhead JL Schiavon S Guariso G Ceroti M Nitti D Clinical relevance of Helicobacter pylori cagA and vacA gene polymorphisms Gastroenterology 2008 135 91 99 10.1053/j.gastro.2008.03.041 18474244

[B6] Fukuta K Azuma T Ito Y Suto H Keida Y Wakabayashi H Watanabe A Kuriyama M Clinical relevance of cagE gene from Helicobacter pylori strains in Japan Dig Dis Sci 2002 47 667 674 10.1023/A:1017949026509 11911357

[B7] Oleastro M Cordeiro R Ferrand J Nunes B Lehours P Carvalho-Oliveira I Mendes AI Penque D Monteiro L Megraud F Evaluation of the clinical significance of homB, a novel candidate marker of Helicobacter pylori strains associated with peptic ulcer disease J Infect Dis 2008 198 1379 1387 10.1086/592166 18811585

[B8] Oleastro M Cordeiro R Menard A Yamaoka Y Queiroz D Megraud F Monteiro L Allelic diversity and phylogeny of homB, a novel co-virulence marker of Helicobacter pylori BMC Microbiol 2009 9 248 10.1186/1471-2180-9-248 19954539 PMC2795765

[B9] Peek RM Jr Thompson SA Donahue JP Tham KT Atherton JC Blaser MJ Miller GG Adherence to gastric epithelial cells induces expression of a Helicobacter pylori gene, iceA, that is associated with clinical outcome Proc Assoc Am Physicians 1998 110 531 544 9824536

[B10] Yamaoka Y Kikuchi S el-Zimaity HM Gutierrez O Osato MS Graham DY Importance of Helicobacter pylori oipA in clinical presentation, gastric inflammation, and mucosal interleukin 8 production Gastroenterology 2002 123 414 424 10.1053/gast.2002.34781 12145793

[B11] Yamaoka Y Ojo O Fujimoto S Odenbreit S Haas R Gutierrez O El-Zimaity HM Reddy R Arnqvist A Graham DY Helicobacter pylori outer membrane proteins and gastroduodenal disease Gut 2006 55 775 781 10.1136/gut.2005.083014 16322107 PMC1856239

[B12] Parkin DM The global health burden of infection-associated cancers in the year 2002 Int J Cancer 2006 118 3030 3044 10.1002/ijc.21731 16404738

[B13] Ferlay JBF Pisani P GLOBOCAN 2002: cancer incidence, mortality and prevalence worldwide, Version 2.0.IARC CancerBase no 5 2004 Lyon: IARC Press http://www-dep.iarc.fr/

[B14] Nguyen LT Uchida T Murakami K Fujioka T Moriyama M Helicobacter pylori virulence and the diversity of gastric cancer in Asia J Med Microbiol 2008 57 1445 1453 10.1099/jmm.0.2008/003160-0 19018013

[B15] Yamaoka Y Kato M Asaka M Geographic differences in gastric cancer incidence can be explained by differences between Helicobacter pylori strains Intern Med 2008 47 1077 1083 10.2169/internalmedicine.47.0975 18552463 PMC3732488

[B16] Nguyen LT Uchida T Kuroda A Tsukamoto Y Trinh TD Ta L Mai HB Ho DQ Hoang HH Vilaichone RK Evaluation of the anti-East Asian CagA-specific antibody for CagA phenotyping Clin Vaccine Immunol 2009 16 1687 1692 10.1128/CVI.00200-09 19776193 PMC2772380

[B17] Imagawa S Yoshihara M Ito M Yoshida S Wada Y Tatsugami M Takamura A Tanaka S Haruma K Chayama K Evaluation of gastric cancer risk using topography of histological gastritis: a large-scaled cross-sectional study Dig Dis Sci 2008 53 1818 1823 10.1007/s10620-007-0077-x 17999184

[B18] Uemura N Okamoto S Yamamoto S Matsumura N Yamaguchi S Yamakido M Taniyama K Sasaki N Schlemper RJ Helicobacter pylori infection and the development of gastric cancer N Engl J Med 2001 345 784 789 10.1056/NEJMoa001999 11556297

[B19] Atherton JC Cover TL Twells RJ Morales MR Hawkey CJ Blaser MJ Simple and accurate PCR-based system for typing vacuolating cytotoxin alleles of Helicobacter pylori J Clin Microbiol 1999 37 2979 2982 10449485 10.1128/jcm.37.9.2979-2982.1999 PMC85427

[B20] Gerhard M Lehn N Neumayer N Boren T Rad R Schepp W Miehlke S Classen M Prinz C Clinical relevance of the Helicobacter pylori gene for blood-group antigen-binding adhesin Proc Natl Acad Sci USA 1999 96 12778 12783 10.1073/pnas.96.22.12778 10535999 PMC23096

[B21] Ikenoue T Maeda S Ogura K Akanuma M Mitsuno Y Imai Y Yoshida H Shiratori Y Omata M Determination of Helicobacter pylori virulence by simple gene analysis of the cag pathogenicity island Clin Diagn Lab Immunol 2001 8 181 186 11139216 10.1128/CDLI.8.1.181-186.2001 PMC96031

[B22] Yamaoka Y Kodama T Gutierrez O Kim JG Kashima K Graham DY Relationship between Helicobacter pylori iceA, cagA, and vacA status and clinical outcome: studies in four different countries J Clin Microbiol 1999 37 2274 2279 10364597 10.1128/jcm.37.7.2274-2279.1999 PMC85136

[B23] Chiba T Seno H Marusawa H Wakatsuki Y Okazaki K Host factors are important in determining clinical outcomes of Helicobacter pylori infection J Gastroenterol 2006 41 1 9 10.1007/s00535-005-1743-4 16501851

[B24] Yamaoka Y Orito E Mizokami M Gutierrez O Saitou N Kodama T Osato MS Kim JG Ramirez FC Mahachai V Helicobacter pylori in North and South America before Columbus FEBS Lett 2002 517 180 184 10.1016/S0014-5793(02)02617-0 12062433

[B25] Oleastro M Cordeiro R Yamaoka Y Queiroz D Megraud F Monteiro L Menard A Disease association with two Helicobacter pylori duplicate outer membrane protein genes, homB and homA Gut Pathog 2009 1 12 10.1186/1757-4749-1-12 19545429 PMC2706848

